# *Isotria medeoloides*, a North American Threatened Orchid: Fungal Abundance May Be as Important as Light in Species Management

**DOI:** 10.3390/plants10091924

**Published:** 2021-09-15

**Authors:** Dennis Whigham, Melissa McCormick, Hope Brooks, Brian Josey, Robert Floyd, Jason Applegate

**Affiliations:** 1 Smithsonian Environmental Research Center, 647 Contees Wharf Road, Edgewater, MD 21037, USA; mccormickm@si.edu (M.M.); brooksh@si.edu (H.B.); 2 Center for Environmental Management of Military Lands (CEMML), Colorado State University, Fort A. P. Hill, VA 22427, USA; brian.w.josey.ctr@mail.mil; 3 Headquarters Department of the Army, Washington, DC 20001, USA; robert.h.floyd4.civ@mail.mil; 4 Natural Resources Branch, Fort A. P. Hill, VA 22427, USA; jason.r.applegate.civ@mail.mil

**Keywords:** Orchidaceae, *Isotria medeoloides*, orchid mycorrhiza, canopy thinning, management

## Abstract

The management of endangered or threatened plant species is difficult if protocols are not developed to propagate species for the purpose of restoration or the enhancement of existing populations. The management of endangered and threatened orchids is especially difficult because of the obligate interactions between orchids and orchid mycorrhizal fungi. *Isotria medeoloides* is a federally threatened forest-dwelling orchid species with a wide distribution in eastern North America. Seeds have not been successfully germinated and current management is based primarily on using subcanopy thinning to increase light in areas where monitoring demonstrates that populations are declining. We report the results of long-term monitoring efforts, canopy thinning, and orchid mycorrhizal fungus abundance studies at two locations in Virginia. The declining populations responded positively to the experimental and natural thinning of the canopy. At one site, the response was the result of understory canopy thinning. At the second site, the response was due to the natural death of a canopy tree. In light of the dramatic increase in fungal abundance following death of the canopy tree, we propose the *Fungal Abundance Hypothesis* as an additional approach to the management of endangered plant species. The removal of canopy trees in or adjacent to *Isotria* populations results in an increase in dead belowground biomass (i.e., roots of the dead canopy tree) that provides substrates for microbial growth, including orchid mycorrhizal fungi, that benefit *Isotria*.

## 1. Introduction

The management of threatened plant species requires knowledge about the ecology of the species, including the monitoring of the species and the environmental factors in ecosystems where the species occur [1]. Orchidaceae species represent a particular challenge because of the large number of globally threatened species and the difficulty of developing successful recovery plans, when basic information on the critical elements of orchid life cycles is frequently lacking [2,3]. Approaches to the management of orchids vary from protecting their habitat, especially global hot-spots with a high species diversity [4,5], to propagating orchids from seed for restoration purposes [3]. The management of existing populations presents another challenge because of threats from invasive species [6] or changes in ecosystem management in orchid habitats, such as grasslands [7] and forests. In the eastern US, fire suppression in forests has had a major impact on the characteristics of plant communities [8]. In some instances, orchid populations are threatened by the increased abundance of native species, especially herbivores [9].

Globally, all orchids have received some protection by their inclusion in the CITES Convention on International Trade in Endangered Species of Wild Fauna and Flora. Some countries have provided further protection through the implementation of laws and regulations [10]. In the USA, a goal of the USA Fish and Wildlife Service is to improve the status of endangered or threatened species under the Endangered Species Act (ESA) to the point where protection is no longer required because a species has recovered. A critical component of the ESA is the development of recovery plans for listed species that include specific recommendations for their management, including criteria to monitor recovery. However, the meaning of ‘recovery’ is not always straightforward [11]. Examples of criteria used to monitor recovery include the number of populations and the sizes of populations over time [3].

Recovery plans for native plants involve a variety of approaches [3], including the protection of habitats where species occur, the monitoring of plants to track population changes over time, and conducting research that will support propagation and restoration efforts. Recovery plans almost always include descriptions of the research needed to understand the life history requirements of species. Some recovery plans include the use of Population Viability Analyses (PVA) but most of those efforts need a broader set of relevant ecological and environmental data than is available in order to be effective [12]. Zeigler et al. [12] analyzed more than 250 species recovery plans and more than 220 publications that described plant PVAs. Most publications that featured PVAs as part of their recovery plans used matrix models with less than five years of demographic data, which included little of the information that would be needed to fully assess the success of recovery plans. They concluded that successful PVAs require long-term data in order to capture the variations in populations’ vital rates that are used in matrix models. To be successful, recovery plans should result in research and monitoring that guide successful restoration efforts. One example of a recovery plan and subsequent research that is guiding restoration efforts is the plan for the endangered *Platanthera leucophaea* (Eastern Prairie Fringed Orchid). A species recovery plan was published [13] following efforts to assess the status of its populations [14]. Soil characterization in habitats where the species occurred was an element of the species recovery plan [15]. Important life history topics were addressed, including the development of successful protocols for seed germination and the identification of the orchid mycorrhizal fungi associated with all life history stages [16,17,18]. The monitoring and research efforts are currently guiding efforts to establish populations in areas where the species had previously been extirpated [Larry Zettler, personal communication].

In the USA, of the approximately 210 orchid species [19], 15 are listed as endangered or threatened at the national level. Almost all native orchids, however, have been listed in one or more categories of threat in individual states of the USA and Canadian provinces [19]. One of the 15 federally threatened or endangered orchids is *Isotria medeoloides* (Small Whorled Pogonia), hereafter referred to as *Isotria*. Unlike *P. leucophaea*, attempts to germinate *Isotria* seeds for the development of propagation protocols have not been successful, even though several attempts to germinate mature and ‘green pod’ seeds have been conducted across several institutions. The successful management of *Isotria* depends on developing protocols for maintaining or increasing the size of known populations, ideally through the use of enhanced flowering and seed production to increase the possibility of new plant recruitment, or through the emergence of dormant plants [20]. The most recent species recovery plan for *Isotria* was published in 2008 [21], and is currently being updated [Cherry Keller, USFWS, personal communication]. One element of the *Isotria* recovery plan, based on research by Brumback et al. [22] was the performance of experiments to increase the light in the forest understory, especially in the southern parts of the species range, where populations are almost always smaller [21].

The manipulation of light levels has been used as an approach in plant-based management as light levels often decrease during ecosystem maturation, causing understory plants to decline in the absence of management [23,24,25,26,27]. Here we present data from ongoing long-term monitoring studies of populations of *Isotria* at two sites in Virginia. We have three goals. The first is to present the results of a recommended regional test of a management approach, understory canopy thinning, that had previously been successfully applied to populations in New England [20,28]. Additional canopy thinning experiments were recommended because it was not known if a similar approach would be successful for populations in the southern portion of the species range, where climatic conditions differ and where populations are smaller compared to the larger populations in the northern part of the range. The second goal is to compare the response of plants in a population where a ‘natural’ canopy thinning experiment occurred when a canopy tree died and fell at the site of a colony where the number of plants had declined to two. The previous thinning experiment in New England [20,28] and the ongoing experiment that we are conducting in Virginia both focused on increasing understory light through the removal of small understory trees and shrubs. The ‘natural’ canopy thinning experiment provided an opportunity to quantify a population response to the death of a canopy tree that potentially resulted in a higher level of light in the understory. Third, we compared the abundance of Russulaceae orchid mycorrhiza DNA associated with *Isotria* that was present in the soil adjacent to plants that had emerged from dormancy prior to the ‘natural’ tree death [29] with the abundance of DNA in the soil adjacent to plants that emerged subsequent to the death of the canopy tree. We previously found that the soil adjacent to plants that emerged from dormancy had more orchid mycorrhiza DNA compared to the soil adjacent to plants that had not emerged from dormancy [29]. The death of a large canopy tree, in addition to providing higher light levels in the understory, also generated a large amount of belowground biomass available for decomposition, potentially resulting in an increase in the abundance of orchid mycorrhiza associated with *Isotria*, in turn resulting in more plants emerging from dormancy. In view of the results of our DNA comparison, we propose that the abundance of orchid mycorrhizal fungi in the soil adjacent to orchids (referred to as the *Fungal Abundance Hypothesis*) should be considered an element of the management of native orchids.

## 2. The Species

*Isotria medeoloides* is one of two species in the genus *Isotria* that are endemic to Eastern North America; both species have overlapping ranges and occur in forests. *Isotria*’s historic and extant distribution is from Maine to Georgia in the eastern USA and west as far as Missouri. Ontario is the only Canadian province where the species is known to occur [30]. The largest number of known populations are in New England (see the website of NatureServe, http://www.natureserve.org (accessed on 13 September 2021)), but southeastern states of Virginia, North Carolina, South Carolina, and Georgia have significant numbers of populations. Nationally, *Isotria* was initially listed as endangered, but its status changed to threatened in 1994, when a minimum number of known sites were found to have viable populations in permanently protected sites (Final Rule to Reclassify the Plant *Isotria medeoloides* (Small-whorled Pogonia) from endangered to threatened (Federal Register. Thursday, 6 October 1994)). *Isotria* is endangered in Canada [30], threatened in Georgia and New Hampshire, and endangered in the other states of the USA where it has been evaluated (https://goorchids.northamericanorchidcenter.org/species/isotria/medeoloides/ (accessed on 13 September 2021)).

**Figure 1:**
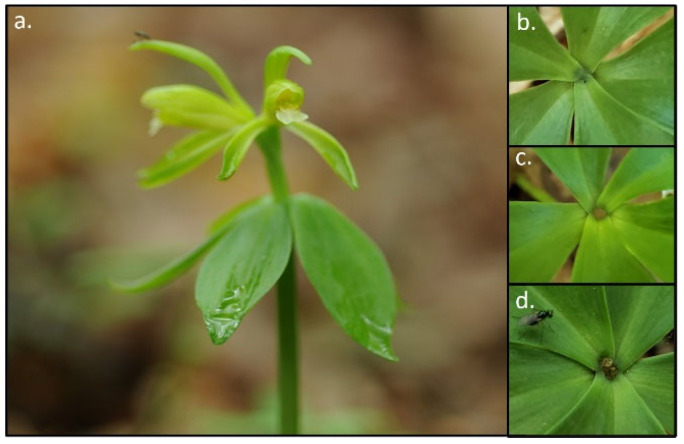
Flowering plant of *Isotria medeoloides*. (**a**) Leaves, typically 5, are in a whorl at the top of the stem, which emerges in the spring. Most reproductive plants produce one flower but two flowers per plant are not uncommon and, at times, plants with three flowers have been seen. Flowering that does not produce fruit can still be assessed by comparing the scars in the center of the leaf whorl to determine if the plant had 0 (**b**), 1 (**c**), or 2 (**d**) flowers.

Individual vegetative and flowering plants emerge in the spring, and almost all plants have five leaflets in a whorl located at the top of the shoot (Figure 1). Sexually reproductive plants produce one, two, and, rarely, three flowers that are largely self-pollinating [20,31,32], and few potential pollinators have been identified [33]. There is more genetic diversity within populations compared to variation among populations across the range of distribution, and the highest levels of diversity among populations occur in the northern part of the range [34] (M.K. McCormick, unpublished data). Individual plants are perennial, and dormancy occurs for varying numbers of years [20,29,35,36]. Like all orchids, *Isotria* associates with mycorrhizal fungi, and fungi in the Russulaceae have been identified from roots [37]. Fungi in the Russulaceae are considered obligately ectomycorrhizal with plants, and some can simultaneously form mycorrhiza. For *Isotria*, this means that the species is indirectly obtaining resources from trees through the fungi. As noted in the Introduction, Rock-Blake et al. [29] found that *Isotria* population dynamics, including dormancy, are related to the abundance of Russulaceae fungi.

Long-term monitoring of *Isotria* has occurred at a several sites [20,28,38], but few long-term data have been published in peer-reviewed articles since Mehrhoff’s studies [35,36]. Brumback et al. [22] and Brumback [28] reported monitoring data as part of an experiment to determine if the decline of *Isotria* populations in New Hampshire was the result of increased shading as forests matured. The removal of understory trees and shrubs resulted in a significant increase in light levels in plots where *Isotria* was monitored, compared to controls. The number of plants and the number of fruits produced annually increased in plots where the subcanopy was thinned. Brumback et al. concluded that canopy thinning could be a management option for *Isotria* populations but noted that their research was not replicated. Dibble et al. [20] reported the results of the long-term monitoring of populations in Maine, which included a canopy thinning experiment. The results from the Maine study were similar to those reported by Brumback [28] and stressed the importance of management that enhances fruit production.

## 3. Results

**Figure 2:**
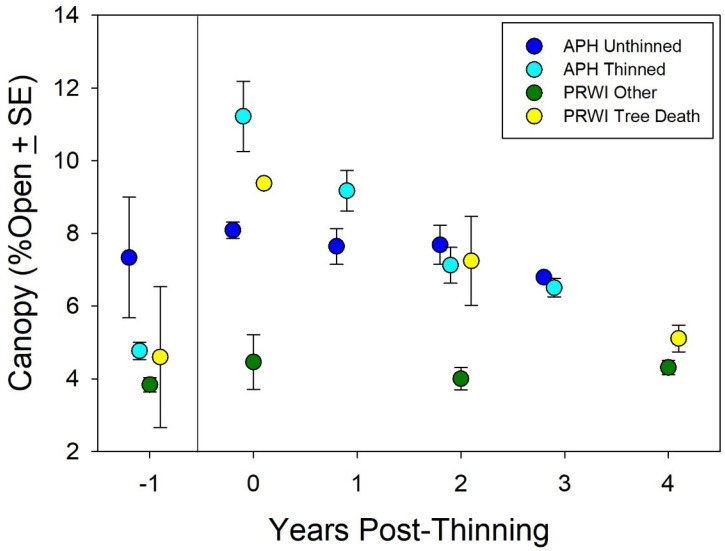
Graph of mean canopy openness (± standard error across multiple colonies and multiple image locations in each colony) at *Isotria* population locations before and after canopy thinning (APH) and the death of a canopy tree (PRWI). ‘PRWI Other’ refers to populations at PRWI where no canopy tree died.

At Fort A.P. Hill (hereafter referred to as APH) and Prince William Forest Park (hereafter referred to as PRWI), both thinning and tree death increased canopy openness. The effect of the thinning experiment at APH and the death of the canopy tree at PRWI on canopy openness is shown in Figure 2. The canopy openness was higher at APH than at PRWI prior to the thinning experiment at APH and the death of the canopy tree at PRWI. At both sites, the canopy openness increased by about 35% following thinning. The canopy openness was highest at the time of the experimental thinning at APH and at the time the canopy tree died at PRWI (Year 0 on the x-axis in Figure 2). At both sites, the canopy openness began to decline over time. This resulted in a significant Thinning effect (z-value = 2.13, *p* = 0.03), but not a significant Relative Year effect (z-value = 1.92, *p* = 0.052) or Thinning χ Relative Year interaction (z-value = −0.54, *p* = 0.59).

**Figure 3:**
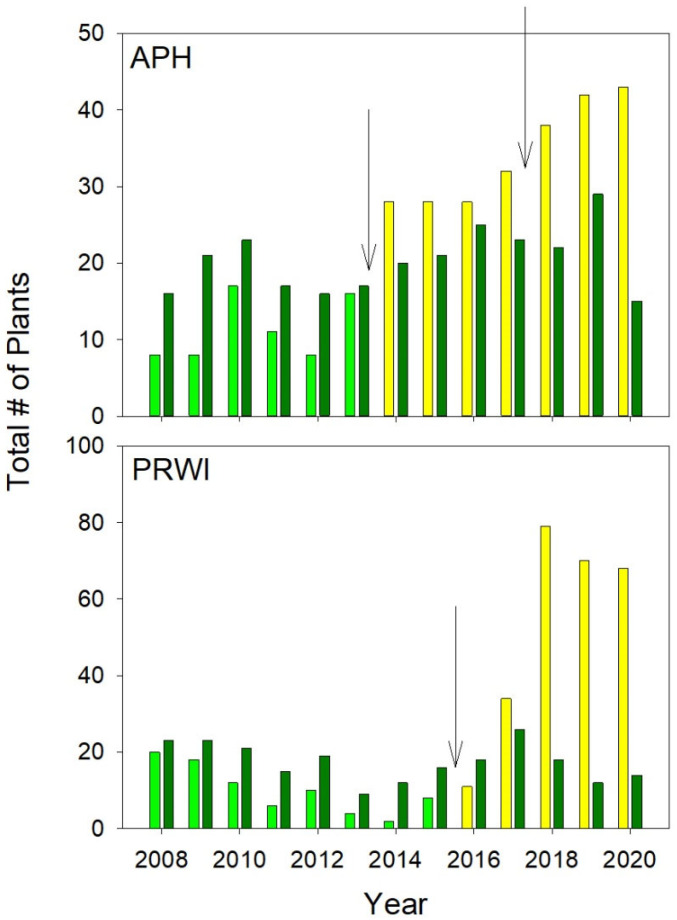
Changes in the number of plants in *Isotria* populations at Fort AP Hill (APH) and Prince William Forest Park (PRWI) in response to Thinning (APH) and the death of a canopy tree at the edge of the population (PRWI). Dark green bars = the number of plants in all non-thinned populations. Light green bars = the number of plants in ‘thinned’ populations before the Thinning experiment began. Yellow = the number of plants in ‘thinned’ populations after the experiment began. The arrows indicate when Thinning (APH) or tree death (PRWI) occurred.

The effect of Thinning or tree death on the total number of emergent plants (Figure 3), indicated as the interaction between Thinning and Relative Year in the GLM with poisson distribution, was significant (z = 9.0, *p* ≤ 0.001), while neither the Thinning (z = 0.33, *p* = 0.74) nor Relative Year’s (z = 0.47, *p* = 0.64) main effect was significant. At APH, the number of emergent plants in un-thinned colonies varied from year to year (Figure 3), with no clear trend. The number of plants in the thinned colonies also varied from year to year prior to the Thinning, but an upward trend began following the first Thinning in 2013. In every year after 2013, the number of plants in the thinned populations was higher than the number of plants in the control plots; the opposite relationship was seen between 2008 and 2013, prior to Thinning. The same pattern was observed at PRWI. The number of plants in the population where the canopy tree died was initially lower than the number of plants in the control plots and, by 2014, the number of plants in the population where the tree would later die had declined to two emergent plants. The number of plants at the site where the tree died started to increase in 2015, when the canopy tree began to decline, and by 2017 the number of plants at that site was higher than that of the controls. The differences between the site where the canopy tree died and the controls was dramatic from 2018 onwards. By 2018, the number of plants at the site where the tree died was higher than in any other colony monitored at the two sites.

**Figure 4:**
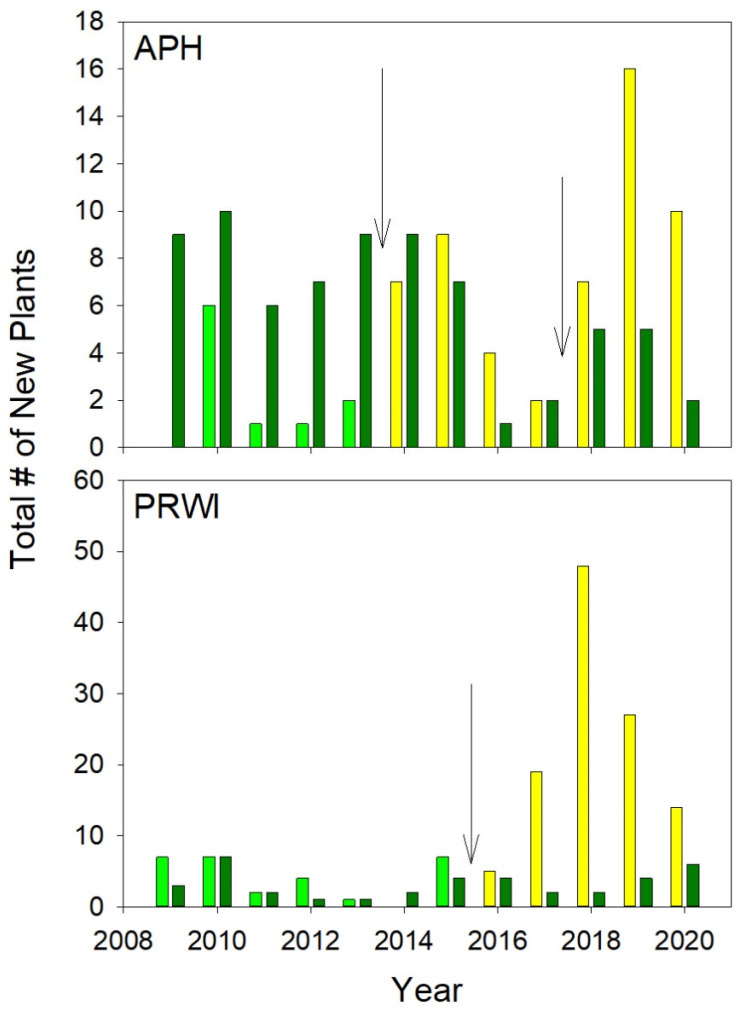
Number of new plants at APH and PRWI. Color codes and arrows are as shown in Figure 3 legend.

The number of new plants that appeared annually (Figure 4) followed the same trends as the total number of plants shown in Figure 3. The number of new plants at APH varied annually, from a high of 10 to a low of 1, in the un-thinned populations prior to the Thinning (Figure 4). The number of new plants in the thinned populations also varied annually but was very low prior to the Thinning. Beginning in 2016, and with the exception of 2017, the number of new plants was higher in the thinned plots than in the controls. The number of new plants at PRWI also varied annually (Figure 4) but the increase in new plants at the colony where the tree died was dramatic from 2017 onwards, and new plants accounted for most of the total plants shown in the population (Figure 3). This was indicated by a significant Thinning–Relative Year (z = 2.7, *p* = 0.007) interaction. Neither the Thinning nor Relative Year’s main effect was significant.

**Figure 5:**
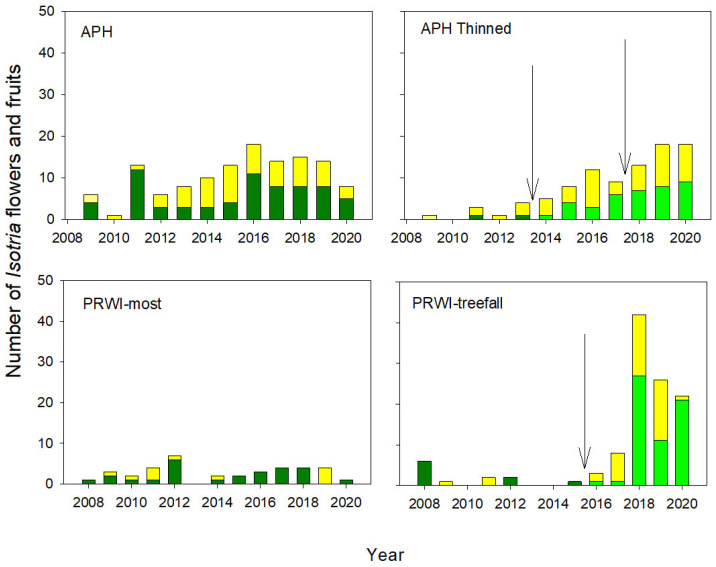
Flowering (green) and fruiting (yellow) in non-thinned (graphs on the left) and thinned (APH) areas, or where a canopy tree died at PWRI (graphs on the right). Color codes and arrows are as described in the legend of Figure 3.

The changes in the number of flowers and fruits following the increase in canopy openness were also significant (Thinning x Relative Year, flowering: z = 4.9, *p* < 0.001; fruiting: z = 2.3, *p* = 0.02) and were most dramatic at PRWI (Figure 5). The number of fruits relative to flowers varied annually in the controls at both sites but there was a steady increase, beginning in 2017, in the number of flowering plants following the increase in canopy openness.

**Figure 6:**
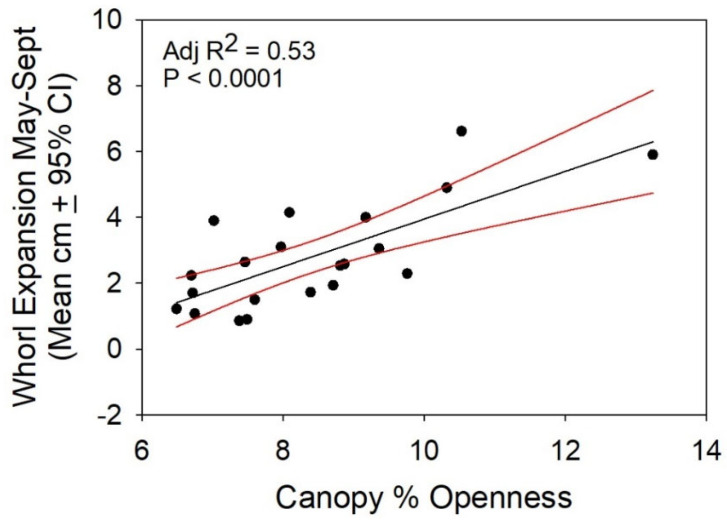
Within-year colony mean whorl expansion as a function of canopy openness. Each point is the mean for a population in a year. The red lines indicate 95% confidence intervals about the regression of whorl expansion on canopy openness. Plants that were growing at sites with more light grew more between May and September.

**Figure 7:**
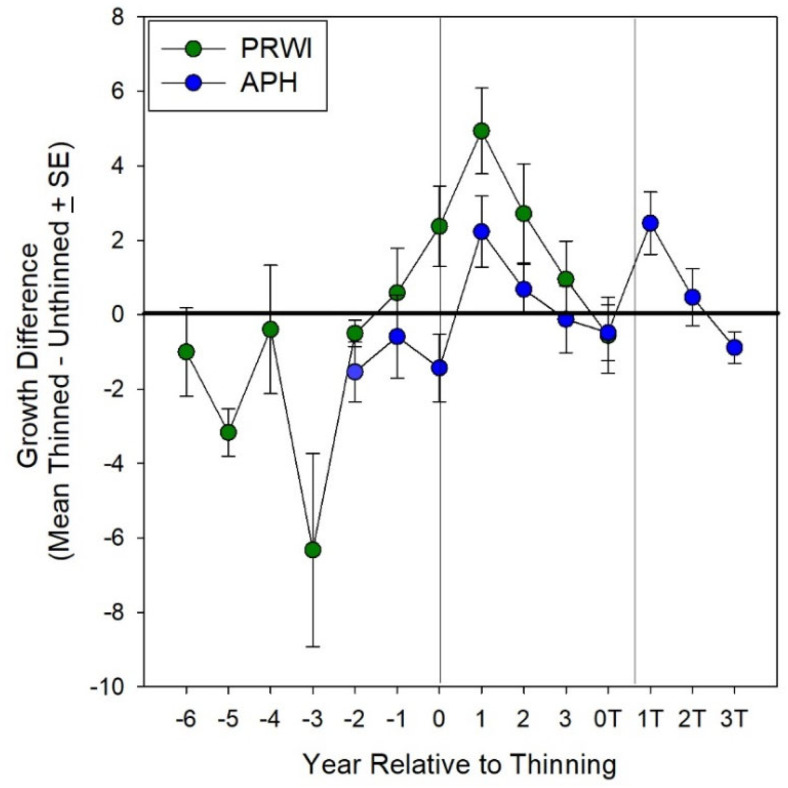
Comparison of plant growth in colonies at Prince William Forest Park (PRWI, green) and Fort A.P. Hill (APH, blue), showing the difference in growth (mean difference between growth ± 1SE) between thinned (or tree death) and un-thinned populations, relative to the year of thinning (treefall for PRWI). The years relative to thinning 0T-3T refer to years 1–3 after a second thinning in these colonies.

The within-year growth of *Isotria* leaf whorls was related to canopy openness (adj R^2^ = 0.53, *p* < 0.001; Figure 6), and the mean year-to-year growth of the leaf whorls of plants at both sites in response to the increased canopy openness is shown in Figure 7 (Thinning × Pre/Post Thinning: F = 19.2, *p* = 0.02). The main effects of Thinning (*p* = 0.81), Pre/Post-Thinning (*p* = 0.46), and colony within Thinning (*p* = 0.66) were not significant. Figure 7 is a comparison of the differences in the growth of plants before and after the increase in canopy openness. The negative values indicate that the growth of plants in control populations (where no Thinning occurred) was greater than it was in areas where canopy openness increased. The positive values show the opposite response: growth was greater for plants in populations where canopy openness increased. The growth of plants in control colonies at both sites was greater prior to the increase in canopy openness, with the exception of PRWI the year before zero, when canopy openness increased due to the slow death of the canopy tree. The increased leaf growth of plants at PRWI was greater than the increased growth of plants at APH and the growth response continued for five years.

**Figure 8:**
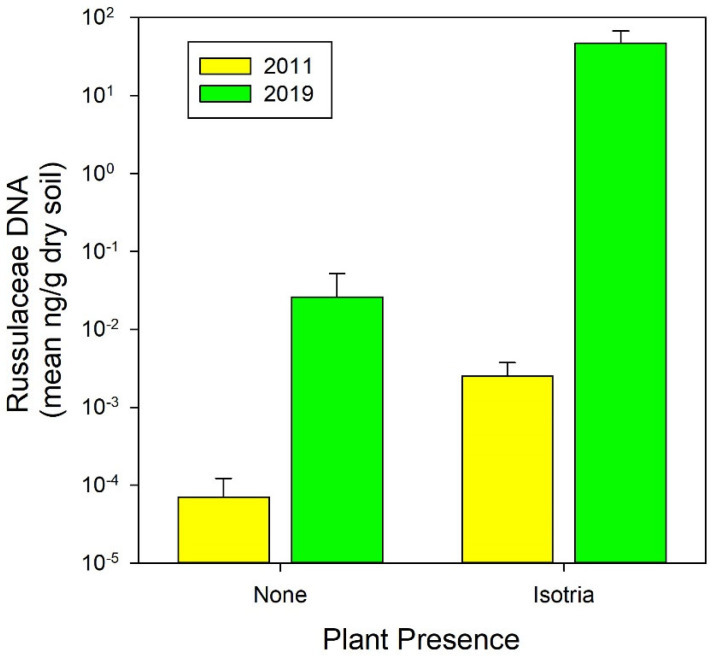
Concentration of Russulaceae DNA in soil samples collected in the “natural experiment” population, where a tree died and fell, in areas where no plants occurred (left bars) and areas where plants emerged (right bars) in 2011 (yellow bars, data from [29]) and 2019 (green bars). Note the log scale on the y-axis.

The analysis of the soil samples from the PRWI population where the canopy tree died demonstrated that the abundance of Russulaceae DNA in 2019 both in the soil adjacent to the emergent plants and in the soil without any detected emergent plants was more than four orders of magnitude greater than that measured by Rock-Blake et al. [29], at the same site, in 2011 (Figure 8; Study: F = 39.2, *p* < 0.001; Orchid vs. Bulk Soil: F = 28.8, *p* < 0.001; Study χ Orchid vs. Bulk Soil: F = 0.003, *p* = 0.98). Notably, the concentration of Russulaceae DNA in the soil samples associated with unoccupied areas in 2019 was greater than its concentration in soil associated with emergent *Isotria* in 2011, suggesting that many previously unoccupied areas may now have enough Russulaceae fungi to support *Isotria*.

## 4. Discussion

Multiple factors influence the ecology of woodland herbs [39,40,41,42] but light [43], whether too much [44] or too little [45,46], is clearly important, indicating that the manipulation of light levels reaching the forest understory is a potential management option for species restricted to forests, and it has been recommended for *Isotria* [21].

Brumback et al. [22] and Dibble et al. [20] found that the number of plants monitored in the New Hampshire and Maine populations had declined. After conducting canopy thinning studies, *Isotria* in areas that received increased light responded positively. At the New Hampshire site, there was an almost ninefold increase in the number of plants and an increase in the number of fruits from 4 or fewer per year to an average of almost 10 and a maximum of 21. The number of plants continued to increase for almost 20 years, and only in 2018–2019 did the total number of plants start to decline [28]. The number of flowering plants also increased steadily over almost 20 years, with a potential downward trend in fruit production starting in 2018–2019. In the 2019 publication Brumback also reported the results of thinning the canopy at a second population in New Hampshire in 2012 (Group A in [28]). The monitoring of plants in the treated population had begun in 1982 and the population had been declining steadily, reaching a low of 26 plants in 2009, before the canopy was thinned in 2012. The number of plants in the treated population increased to 54 by 2016, followed by a decline between 2017–2019. Flowering also increased dramatically in the treated population from 1 plant in 2010 to 39 plants in 2015. Dibble et al. [20] reported the results of the monitoring of declining populations of *Isotria* at four sites in Maine from 1986 to 2000. They conducted a canopy thinning study in 1993 and found a short-term positive effect on the number of plants, the plant size, and the number of fruits produced. They concluded that the main focus of *Isotria* management should be on the creation of conditions that result in the production of fruits because it is important to assure a supply of seeds in order to increase the possibility of plant establishment in the future, in response to either natural conditions or the effects of human activities. The studies in New Hampshire and Maine provide insight into how more southern populations might be managed, but until the studies were conducted at APH the responses of the southern populations to canopy thinning, which are typically smaller, were unknown.

On two occasions among three colonies of *Isotria* at APH, understory thinning resulted in increased canopy openness (Figure 2). The three colonies responded to this increased canopy openness with an increase in the total number of plants (Figure 3), the appearance of new plants (Figure 4), increased fruit production (Figure 5), and increased growth (Figure 7). Continued monitoring will enable us to determine whether the benefits of increased canopy openness will persist for as long as the responses that Brumback [28] reported for the New Hampshire populations, or whether they will be short term, as reported by Dibble et al. [20]. Current trends suggest the latter.

In addition to canopy thinning, other approaches, such as burning and grazing, have been applied in a variety of ecosystems, with benefits for orchids [47]. In some instances, orchids have been the focus of directed management activities designed to increase population size or stop population decline [48,49,50,51]. However, management whose only goal is to increase light levels may not be the most appropriate strategy because several factors may interact to influence plants’ performance. Dibble et al. [20] found that increased canopy openness resulted in the increased growth of plants, especially ferns, that would compete with *Isotria* and increased herbivory that has been shown to have a negative impact on native orchids [9,52]. The increased competition was not a problem at our study sites because few other herbs were present and none that were there were dense enough or high enough to interfere with *Isotria*. In addition, herbivory was not a problem because all of the plants were protected by cages that were specifically designed to eliminate deer herbivory without reducing light. Thomas et al. [52] used a combination of thinning and fertilization to examine the responses of 54 species of understory plants in Douglas-fir dominated forests in Washington. All but seven of the species, including the orchid *Listera cordata*, benefited from canopy thinning. By contrast, only 17 species responded positively to the fertilization treatment, and *Listera cordata* was not one of them. Falb and Leopold [49] studied the effects of canopy thinning on *Cypripedium candidum* in a partially drained New York fen. Larger plants and plants that flowered were associated with higher light conditions; however, the overall response to the removal of woody vegetation over a 5-year period resulted in a decrease in the total number of plants but an increase in the number of flowering plants. Falb and Leopold found that light levels reaching individual plants were high enough for plants to flower but, at the systemic (i.e., fen) level, light levels did not differ between the treated and control areas. Instead, other factors drove population responses. Falb and Leopold also found that precipitation had a confounding effect on plant performance. Ditching caused the water table to lower and invasion by woody plants, resulting in a lowering of soil moisture that influenced plants equally in treated and untreated areas.

Increased light is not always beneficial and management decisions should give consideration to local conditions rather than broad-scale assumptions [51]. In the case of orchids, however, consideration also needs to be given to the potential impacts of management on orchid mycorrhizal fungi, an essential component of the life history of all orchids and a factor that needs to be considered in all efforts to conserve native orchids [3].

A key to the long-term trend in the number of plants in the thinned colonies at APH will be the relationship between the number of new plants that continue to emerge annually and the number of plants that either die or enter dormancy. Dormancy is an important feature of the life history of *Isotria* [20,22,28,29,35,36]. Brumback found that the number of plants entering dormancy was lower in the thinned population compared to the un-thinned populations [28]. The very large increase in plants at PRWI following the death of the canopy tree (Figure 3) may be related to dormancy as well as reproduction from seeds. We do not know the origin of the new plants that appeared at PRWI (Figure 3). Some may have appeared due to the emergence of plants from dormancy [29], although this would have taken longer than the projected maximum 9-year dormancy previously observed for *Isotria* [21] (and unpublished data from the authors’ ongoing monitoring studies at APH and PRWI); others may have grown following seed germination; still others may have emerged through a combination of both factors.

Rock-Blake et al. [29] found that the emergence from dormancy in *Isotria* was related to the abundance of Russulaceae fungus DNA in the soil. The increased abundance of Russulaceae may also be responsible for the increased germination of seeds and growth of protocorms and seedlings. While there are no data to support this assumption, *Isotria* seeds have been found to remain viable for decades in the soil, and an increase in fungal abundance could potentially trigger germination. *Isotria* seeds in soil in seed packets [53] at the New Hampshire site studied by Brumback et al. remained viable for at least 13 years [D. Whigham, personal observation], and our ongoing studies in Virginia and West Virginia indicate that seeds in the southern populations also remain viable for long periods of time [M. McCormick and D. Whigham, personal observations]. The increased numbers of flowering and fruiting plants in thinned populations in New Hampshire and Maine and the two sites in Virginia provide clear evidence that one benefit of thinning is that the increased production of seeds that contributes to the establishment of a long-lived seed bank will potentially result in new plants in the future.

As was shown in the two studies in New England [20,28], our research demonstrated the positive effect of thinning on the growth of individual plants (Figure 7), the positive relationship between canopy openness and growth (Figure 6), and the increase in fungal abundance in response to the death of the canopy tree at PRWI (Figure 8). Falb and Leopold [49] showed similarly positive relationships between light and plant size in *Cypripedium candidum*. The relationship between plant performance and light could be obscured by the effect of mycorrhizal fungus abundance. *Isotria*, like many orchids, is partially mycoheterotrophic, relying on fungi for some of the carbon and nitrogen needed for its growth [54,55] (and McCormick et al. Unpublished data). Based on the changes in fungal abundance shown in Figure 8, we hypothesize that the greater growth and increase in the number of new plants at PRWI (Figure 3 and Figure 7), compared to APH, was due to an increase in fungal abundance (i.e., the *Fungal Abundance Hypothesis*). The research on *Isotria* reported by Rock-Blake et al. [29] and similar research on other orchids [56,57,58,59,60] have indicated that the abundance of orchid mycorrhizal fungi is important for sustaining orchid populations. Rock-Blake et al. [29] found fungal abundance to be greater in instances where *Isotria* emerged from above ground, compared to where they were dormant. McCormick et al. [58] found the seed germination of three orchid species to be higher in seed packets closer to established plants, where the abundance of orchid mycorrhizal fungi was also higher. McCormick et al. [59] found the abundance of orchid mycorrhizal fungi associated with *Galearis spectabilis* to be positively related to plant density.

## 5. Conclusions

In summary, the results of the thinning experiment at APH support the findings of Brumback’s studies in New Hampshire [22,28] and research by Dibble et al. [20] in Maine, and provide evidence that thinning should be employed more broadly across the range of *Isotria*. Our research also provides novel insights into the potential use of canopy thinning as a management approach in order to increase the abundance of the orchid mycorrhizal fungus in the soil that is important to *Isotria*. This could be used to effectively bolster *Isotria* populations. The 2011 data shown in Figure 8 demonstrates the importance of fungal abundance as describe by Rock-Blake et al. [28]. Fungal abundance was greater in soils adjacent to plants that emerged from dormancy in 2011 compared to the abundance of the fungus in the soil adjacent to plants that remained dormant. The 2019 data demonstrated a dramatic increase in fungal abundance in the soil adjacent to plants that emerged and plants that remained dormant. The underlying reasons for the large increase in fungal abundance in 2019 remain to be determined, but a likely factor was the large amount of dead belowground biomass that became a carbon source for belowground microorganisms following the death of the tree. The dramatic response of the population at PRWI following the death of the canopy tree indicates two potential options for managing *Isotria* populations. In populations that are stable, based on long-term monitoring, understory thinning to increase light may be sufficient. For populations that are declining, canopy thinning may be more useful in sustaining populations by possibly increasing the fungal abundance. This could potentially result in the emergence of more dormant plants, the germination of *Isotria* seeds in the soil seed bank, and increased growth of plants. The results of the research at PRWI were, however, based on the death of one tree at one site. Further research is needed to test the *Fungal Abundance Hypothesis* using a replicated experiment that includes measurements of light as well as fungal abundance before, during, and after the death of a canopy tree.

## 6. Materials and Methods

*Study sites*—Fort A.P. Hill is an approximately 30,700 ha military facility located near Bowling Green, VA, with almost 5600 ha of forest habitat that could potentially support *Isotria* populations. *Isotria* has been monitored at APH since 1984, but our dataset began in 2008, when we began monitoring four known populations. By 2020, the number of populations monitored at APH had increased to 22, mostly as a result of surveys of areas that were slated for forest management or construction. Prince William Forest Park is a National Park located near Quantico, VA. PRWI is more than 6000 ha and is mostly composed of forested areas, in which *Isotria* was first found in the 1980s. There are 14 historical sites at PRWI and 7 sites that are currently active (i.e., where plants appear above ground yearly). Our research at PRWI began in 2008.

*Plants*—At both sites, all individuals in known populations are monitored twice yearly, once in the spring and once in the autumn. When a plant is discovered for the first time, it is given a unique identifier (number), and a metal tag with the plant number is placed 10 cm away from the shoot. The metal tag is secured to the ground with either a metal or PVC wire stake. With a ruler as a guide, a second metal or PVC stake is placed at 10 cm on the opposite side of the tag, with the shoot being equidistant between the stakes. Having two stakes that are equal distance from the plant’s location has helped us to locate plants, in order to determine whether they are still alive below ground when a shoot does not appear above ground. Each autumn, when the second yearly census is made, we determine the status of each plant that was present at the time of the spring census. The data recorded are: (1) shoot missing; (2) Shoot senescing or green; (3) Status of the fruit on plants that flowered in the spring (fruit absence or presence and condition: green, sensing, dehisced). For plants that were present at the spring census, we examine the location where the shoot emerged from the soil to determine if an overwintering bud is present or absent. If a bud is present, its diameter is measured. The locations of all plants in each population are determined by measuring the distance from each plant to two permanent PVC pipes that are placed in the ground outside of the population. The distance to the individual plants from the two PVC pipes enables us to map the distribution of the plants and assist in relocating them if their tags are not located during annual monitoring events.

The leaf whorl diameters are measured each time plants are monitored, along with the height of the whorl (the distance from the soil surface to the whorl). If plants flowering, the number of flowers is recorded. In some instances, plants produce flowers that fail to produce a fruit, and flowers are shed by the time we conduct the spring census. To determine the presence or absence of flowering, we examine each plant in order to observe whether a flower or flowers have been produced. This is possible to determine because a ‘scar’ (Figure 1) remains at the center of the leaf whorl, indicating the presence of a flower that has been dropped. When plants are visited in the autumn, we remeasure them and determine the number and status of the fruit (e.g., mature, dehisced). At both sites, upside-down tomato cages with netting placed around the bottom third of the cage are secured over each plant to deter deer from browsing, while allowing pollinator access. Cages are removed at the end of each growing season to allow the accumulation of natural leaf litter.

As described above, at APH we replicated the thinning experiment conducted by Brumback et al. [22] in New Hampshire. We chose three populations, or colonies, for the experiment, and chose three other populations, all monitored for the same length of time, to serve as controls. Our goal was to increase light levels by approximately 30% by cutting understory trees and shrubs. The APH sites have been thinned twice: in 2013 and 2017. Thinning was accomplished by using hand clippers and a chain saw, and the cut material was removed from the site. Only trees and shrubs that formed arbuscular mycorrhizal associations were removed, to prevent the removal of ectomycorrhizal trees that could be supporting mycorrhizal fungi that, in turn, could support *Isotria*. As described below, we measure canopy openness at the APH populations annually. We report the following data for control and thinned colonies: canopy openness, the number of plants, the number of flowers and fruits, and the growth of plants based on the annual measurements of whorl diameter.

For PRWI, we also report data on canopy openness and plant data for all colonies, including one population where the death of a large Red Maple (*Acer rubrum*) over the course of approximately three years resulted in the formation of a canopy gap that was, in effect, a natural thinning experiment. For PRWI colonies, we collected hemispherical canopy photos for every year of the study except for 2017 and 2019, which were missed because the camera was in use at other sites.

*Canopy openness*—Hemispherical canopy images were used to determine the amount of light transmitted by the canopy at the locations of several individuals in each *Isotria* colony at both sites. Hemispherical images were taken in the spring and autumn with a fisheye lens on a Nikon Coolpix 990 or Nikon Coolpix 995 camera, mounted on a tripod and levelled and positioned with the camera at 0.5 m directly above individual plants. Photos were taken at the same location every time, and the locations were initially chosen to span the spatial extent of each colony. Sidelook [61] and ImageJ [62] Hemispherical_2.0 package [63] were used to determine the thresholds to convert the digital color images to black and white, which were then analyzed using Gap Light Analyzer v.2.0 [64] to determine the percentage of canopy openness.

*Orchid mycorrhizal fungus abundance*—For the comparison of fungal abundance in soil following the death of the canopy tree at PRWI, we used two sets of data. The first were data that were part of an earlier study [29], when soil samples from the same sites were collected and processed for fungal abundance. In that study we compared fungal abundance in the soil adjacent to plants that had not emerged from dormancy with the soil adjacent to plants that had emerged form dormancy. The methods used to collect and process the soil samples were described by Rock-Blake et al. [29].

The second data set was part of a study to quantify fungal abundance in soil in the PRWI population where the canopy tree died. We divided the colony into 42 grid squares, each 2 m × 2 m. Using this grid, we collected soil from two settings in June, 2019: (1) Soil adjacent to 29 plants that were part of the long-term monitoring effort; and (2) Soil in grid cells that had never contained plants. The methods used to collect and process the soils were similar to those used by Rock-Blake et al. [29] and McCormick et al. [64]. Briefly, one 2.5 cm × 10 cm soil core was removed 10 cm away from up to two sampled plants in each grid square. Soil was collected from the areas adjacent to two plants in cells with 2+ plants, or one plant if only one orchid was present. For grid squares with no emergent or previously observed orchids, we collected only bulk soil samples. Bulk soil samples were composites of five randomly located 2.5 cm × 10 cm soil cores within each of the 42 grid cells. Soils were lyophilized and ground in a mortar and pestle. DNA was then extracted from a 25 mg subsample, using Qiagen DNEasy Soil DNA extraction kits, according to the manufacturer’s instructions. The DNA concentration in the extracts was quantified using a NanoDrop 2000. Quantitative real-time PCR was conducted on 20 ng of source DNA in 20 µL reactions with primers ITS3-R1a and ITS4, as in Rock-Blake et al. [29]. DNA from a *Russula* sp. fruit was used to generate five quantitative standards (1–0.0001 ng/µL), and to adjust target DNA concentrations to per g of dry soil, as per Rock-Blake et al. [29].

*Statistical analysis*—Analyses were carried out using R package [65] and Systat (v.12 for Windows). We compared the percentage of canopy openness at sites that were thinned (APH) or had a tree die (PRWI) with those that were unmanipulated, using a generalized linear model with beta distribution in a glmmTMB package [66]. Thinning (whether or not the canopy was thinned, including the site where the tree died,) and Relative Year (i.e., the year relative to thinning or tree death and designating the repeated measure) as fixed effects, and with Colony nested within Thinning as a random effect. We also compared the number of emergent plants, the number of flowering plants, the number of fruiting plants, and the number of new plants across thinned and unthinned colonies at each site. Each of these count response variables was analyzed in a separate generalized linear model using an lme4 package [67], with poisson distribution to account for the non-normality of count data. We again used Thinning, Relative Year (designating the repeated measure), and the interaction between Thinning and Relative Year as fixed effects and Colony nested within Thinning as a random effect. Nesting Colony within Thinning allowed us to account for very different conditions and colony sizes. In particular, the most vigorous colonies at APH were not thinned, so the thinned colonies began by performing poorly relative to the un-thinned colonies. Furthermore, because tree death and thinning did not occur during the same years and thinning occurred twice, the annual variation differed among the treatments.

We compared the annual growth (e.g., growth in 2016 = whorl diameter in 2016 − diameter in 2015) of plants before and after thinning using a general linear model with Thinning (yes, including tree death, or no), pre- or post-thinning, Thinning χ Pre/Post as fixed effects, and Colony nested within Thinning as a random effect in Systat. In addition to the annual measurements, we also measured the leaf whorl diameter twice in 2017, in May and September, for all emergent plants at both PRWI and APH, and calculated the difference between the diameter in September and in May, as a measure of continued leaf whorl expansion during the growing season. We related that continued growth to mean percent canopy openness for each colony using a linear regression, with canopy openness as the independent and growth as the dependent variable.

The DNA abundance in soils was compared using ANOVA in Systat, with the Bulk Soil vs. Orchid Study and the interaction of the Soil vs. Orchid χ Study as the fixed main effects. The DNA abundance was log-transformed prior to analysis in order to improve normality, as determined using a Shapiro-Wilk test.

## Data Availability

Data are available from the authors (M.M., D.W.) and from annual reports submitted to Fort A.P. Hill (APH) and Prince William Forest Park (PRWI).
